# Adaptive coding occurs in object categorization and may not be associated with schizotypal personality traits

**DOI:** 10.1038/s41598-022-24127-3

**Published:** 2022-11-12

**Authors:** Anna O. Giarratana, Mariia Kaliuzhna, Stefan Kaiser, Philippe N. Tobler

**Affiliations:** 1 Zurich Center for Neuroeconomics, Department of Economics, University of Zurich University of Zurich, Blümlisalpstrasse 10, 8006 Zürich, Switzerland; 2 Division of Adult Psychiatry, Department of Psychiatry, Geneva University Hospitals, Geneva, Switzerland

**Keywords:** Perception, Psychosis, Schizophrenia, Human behaviour, Object vision

## Abstract

Processing more likely inputs with higher sensitivity (adaptive coding) enables the brain to represent the large range of inputs coming in from the world. Healthy individuals high in schizotypy show reduced adaptive coding in the reward domain but it is an open question whether these deficits extend to non-motivational domains, such as object categorization. Here, we develop a novel variant of a classic task to test range adaptation for face/house categorization in healthy participants on the psychosis spectrum. In each trial of this task, participants decide whether a presented image is a face or a house. Images vary on a face-house continuum and appear in both wide and narrow range blocks. The wide range block includes most of the face-house continuum (2.50–97.5% face), while the narrow range blocks limit inputs to a smaller section of the continuum (27.5–72.5% face). Adaptive coding corresponds to better performance for the overlapping smaller section of the continuum in the narrow range than in the wide range block. We find that participants show efficient use of the range in this task, with more accurate responses in the overlapping section for the narrow range blocks relative to the wide range blocks. However, we find little evidence that range adaptation in our object categorization task is reduced in healthy individuals scoring high on schizotypy. Thus, reduced range adaptation may not be a domain-general feature of schizotypy.

## Introduction

Imagine you are awakened in the middle of the night. Naturally, you reach for your phone to check the time. In the dark, the light of the screen is almost blinding; yet, the same screen luminosity in broad daylight would barely stand out. This differential response to the same stimulus reflects a neural process called adaptation or adaptive coding. Adaptive coding occurs when neurons adjust their firing rate to the inputs that are most likely in the current context. At night, the most likely inputs are on the darker end of the brightness spectrum. The light from the screen is therefore brighter than the expected stimuli and elicits a strong neuronal response. On the other hand, during the day, the brightness of the phone screen falls within the luminosity range currently represented, far from the brightest stimulus present, thus producing a much weaker neuronal response.

Adaptive coding has been extensively described for sensory processing^1–4^ and perception. For example, previous work has shown that when one is adapted to a blurry image, a subsequently presented image will appear sharper than it actually is. Similar effects occur for contrast^5^, orientation^6,7^, surround suppression^8^, and mismatch negativity^9^. Adaptive processes contribute also to functions that are more complex, including reward processing^10–13^, object categorization^14,15^, rule learning^16,17^, and attractiveness^18^ and moral judgements^19^. Thus, adaptive coding pervades many everyday functions and enhances sensitivity to the inputs of these functions by accounting for, and thereby reducing the impact of, properties of the environment or context.

Adaptation can occur to the mean (expressed by a shift in the response function) or the range (expressed by a change in the slope of the response function) of possible inputs. Both forms of adaptation are important, often happen in conjunction, and ensure that one remains sensitive to the most likely inputs. However, previous research focused largely on adaptation to the mean^6–8,20–22^, while less work studied adaptation to the range of possible inputs^1,23^. The relative paucity of research on range adaptation is surprising given that it appears to provide an efficient solution to the problem that the possible ranges of environmental inputs approach the infinite while the number of available neurons and their metabolic capacity are limited^24^.

Recent work has highlighted the importance of adaptive coding in psychosis spectrum disorders. For example, patients with schizophrenia or with first episode psychosis, and healthy individuals with schizotypal traits show a reduction in the adaptive use of contextual information for low-level perceptual^8^, reward^25,26^, and hedonic^27^ processes. Thus, malfunctions in adaptive coding could reflect general changes in computations on the psychosis spectrum^28,29^. However, it remains unknown how general this deficit is and whether it extends to high-level perception.

**Figure 1:**
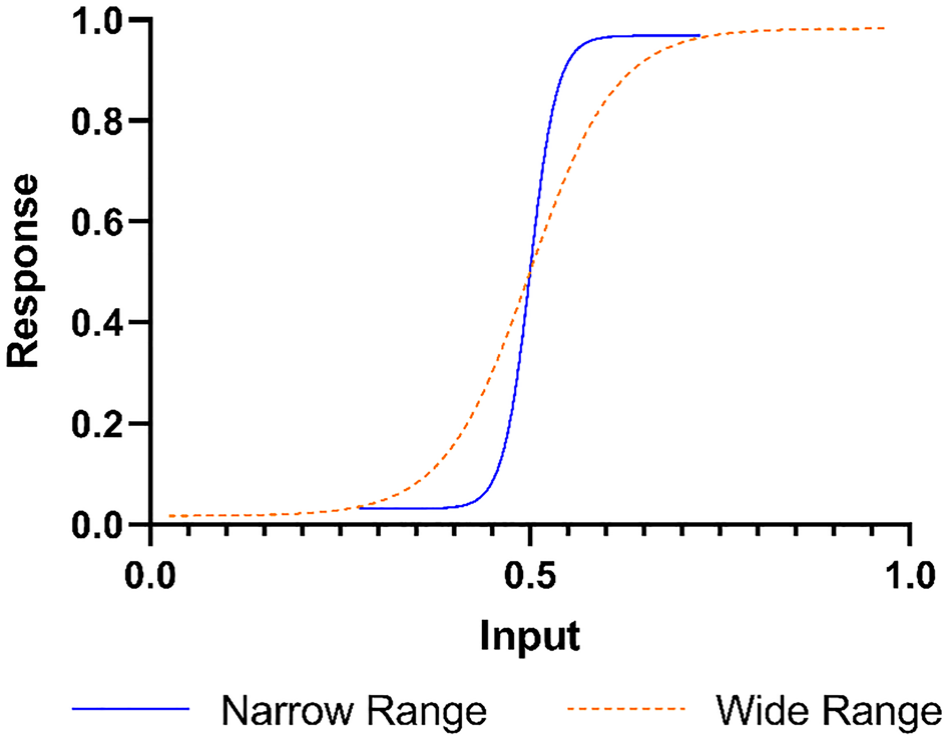
Schematic of range adaptation. With range adaptation, the responses (neural and behavioral output) are adjusted to the most likely input range, resulting in steeper response functions for a narrow range compared to a wide range.

In order to test this, we developed a novel variant of the classic face-house discrimination^30–34^ task to test range adaptation in object discrimination. Participants on the psychosis spectrum viewed morphed images of houses and faces, and had to categorize the morphs as a house or a face. Crucially, the morphs were viewed in either a wide or a narrow range block. In the wide block, morphs ranged from 2.5 to 97.5% face whereas in the narrow block morphs ranged from 27.5 to 72.5% face. We expected participants to show increased accuracy in the narrow range, compared to the wide range, for the morphs presented in both blocks, in line with the hypothesis that adaptation to the range improves sensory discrimination. Under the assumption that adaptive coding to the range does improve sensory discrimination (Fig. 1), in the narrow range, the possible morphs vary less widely, which should allow participants to better differentiate between them, resulting in higher accuracy for the most difficult images. As a first step to explore whether adaptive coding could be an endophenotype of schizophrenia, we also tested whether participants with high schizotypy scores would show reduced range adaptation in object categorization compared to participants with low schizotypy scores.

## Materials and methods

### Participants

We recruited participants from a participant pool administered by the Department of Economics of the University of Zurich. All participants were screened for mental health diagnoses and those with DSM-V Axis I diagnoses were excluded. Data was collected from 87 participants (44F/40M/3O), with a mean age of 24 (ranging from 18 to 35) years (Table 1). Thirty-one participants took part in Experiment 1, 35 in Experiment 2, and 31 in Experiment 3. We used a number of exclusion criteria in order to confirm that participants were paying attention to the task and were motivated to participate. Participants were excluded if they missed any of the attention check questions in the Schizotypal Personality Questionnaire—Brief Revised Updated (SPQ-BRU), if they wrongly answered all of the guided instruction questions, if they exceeded the maximum response time of two seconds more than five times in a block or more than ten times overall, if they had any blocks with an average response time less than 400 ms, or if they had any blocks with an average accuracy of less than 60%. These criteria were determined in initial pilot tests, based on the response patterns of participants who were not engaged in the task. After applying these attentiveness-related exclusion criteria, we excluded one participant from Experiment 1, five participants from Experiment 2, and four participants from Experiment 3 so that 30 participants were included in Experiment 1, 30 in Experiment 2, and 27 in Experiment 3.

**Table 1:** Demographic information for participants in the experiments.

	Experiment #1	Experiment #2	Experiment #3
Age	24.5 (0.681)	23.7 (0.539)	23.9 (0.548)
Gender (F/M/O)	14/15/01	18/11/01	12/14/01
Education in years	5.43 (0.386)	4.81 (0.288)	5.74 (0.379)
SPQ-BRU Total	51.2 (2.13)	43.9 (2.12)	48.3 (2.02)
SPQ-BRU CP	44.8 (2.46)	39.2 (2.24)	38.6 (1.89)
SPQ-BRU IP	54.7 (3.14)	45.6 (2.89)	52.3 (3.12)
SPQ-BRU DO	57.7 (2.81)	49.9 (2.01)	60.3 (3.48)

Mean values (standard error). Gender options are Female, Male, or Prefer Not to Answer. Age is measured in years. Education is the number of years of education after the age of 18. SPQ-BRU subscales are Cognitive Perceptual (CP), Interpersonal (I) and Disorganized (DO).

### Ethics

The project was approved by the Human Subjects Committee of the Faculty of Business, Economics and Informatics (OEC Human Subjects Committee) at the University of Zurich. All participants gave written informed consent to participate in the study in accordance with the Declaration of Helsinki. All methods were carried out in accordance with the relevant guideline and regulations.

### Experimental design and task

#### Face house categorization task

**Figure 2:**
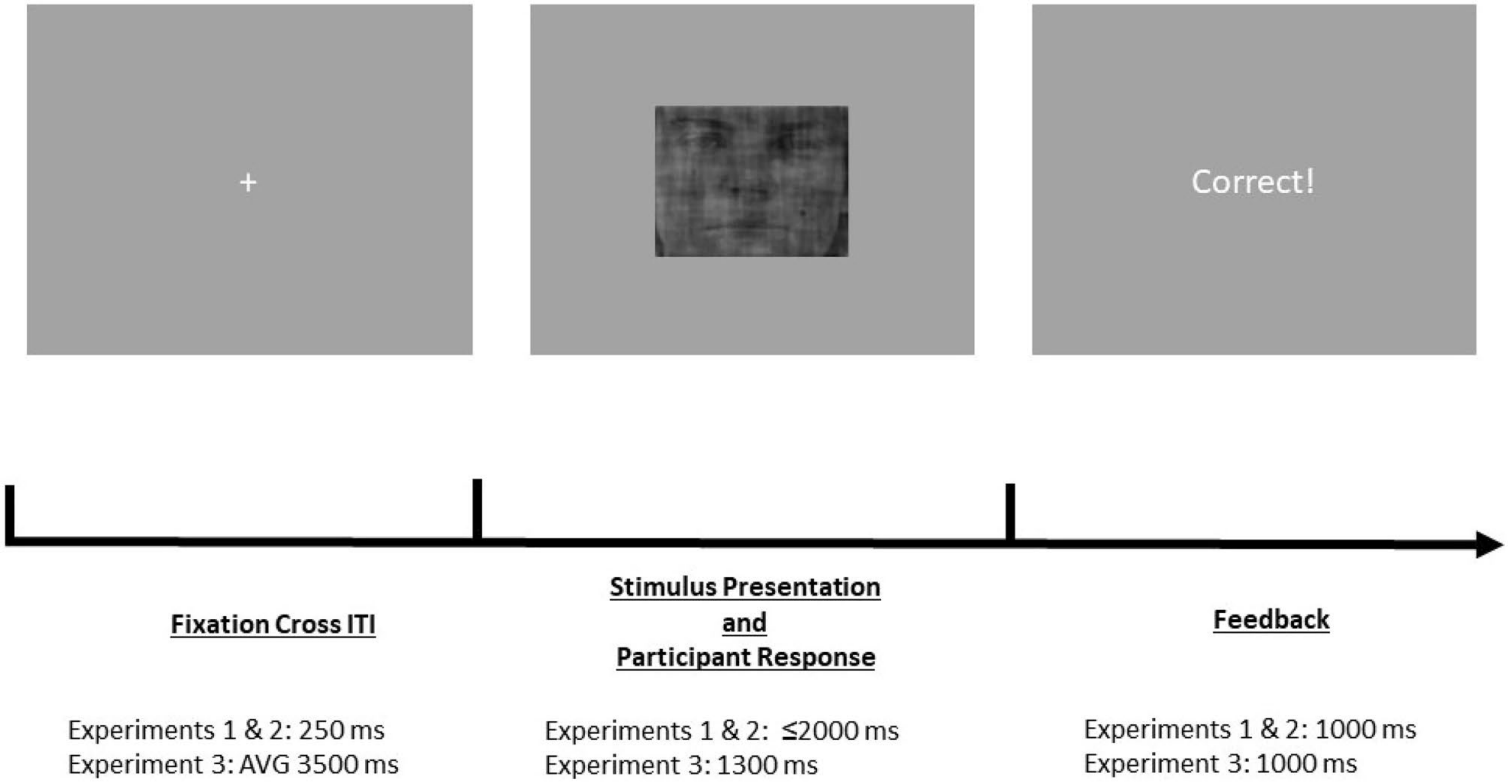
Experimental procedure for the face-house object discrimination task. Example trial of the face-house object discrimination task. We also list the parameters for all three experiments. Participants were shown a fixation cross during the intertrial interval (ITI). In experiments 1 and 2, the ITI lasted a fixed 250 ms. In experiment 3, it lasted an average of 3500 ms, jittered from 1000 to 9000 ms. Following the ITI, participants were shown the stimulus and were instructed to respond whether it more closely resembled a face or a house. In experiments 1 and 2, they were given 2000 ms to respond. Once a response was made, they would move on to the feedback screen. In experiment 3, participants were given 1300 ms to respond. Once a response was made, they remained on the stimulus screen until the 1300 ms had elapsed, at which point they moved on to the feedback screen. The feedback screen was presented for 1000 ms in all three experiments. On the feedback screen, participants were shown either “Correct!” or “Incorrect!” based on whether their response matched the objectively correct answer.

We designed an object categorization task using stimuli and code from Fleming and colleagues^35,36^, available at https://github.com/smfleming/NoisyFaces. Face images were taken and adapted from the Karolinska Directed Emotional Face set. Written consent for the publication of sample images from the Karolinska Directed Emotional Face set exists and can be found at the following location: https://www.kdef.se/home/using%20and%20publishing%20kdef%20and%20akdef.html^37^. London houses were photographed by Fleming and colleagues. We utilized this set of stimuli and code to create morphed images of faces and houses overlaid with a standard white noise matrix at a proportion of 0.35. The stimuli were created using MATLAB Version R2019A (The MathWorks, Natick, USA). In all of the experiments on every trial participants were shown a morph and asked to judge if it resembled more a face or a house. We used the task both online (Experiments 1 and 2) and in the lab (Experiment 3). The online experiments were implemented with the Gorilla Experiment Builder (www.gorilla.sc), the lab experiment with Matlab-based Psychtoolbox Version 3. All experiments used the same basic framework but slightly different parameters in efforts to optimize the task for behavioral measurements and later fMRI data collection (Fig. 2).

#### Experiment 1

In Experiment 1, participants were shown an image for 2.0 s and indicated whether it more strongly resembled a face or a house by pressing one of two buttons. Response keys were counterbalanced across participants. The noise level of 0.35 was determined by initial pilot testing in the lab aiming for an average overall performance of ~ 85%. Following the image, feedback was given for 1.0 s. Trials were separated by an inter-trial interval where a fixation cross was presented for 0.25 s. This experiment included four blocks, two narrow range blocks with morph percentages from 27.5 to 72.5% face, and two wide range blocks with morph percentages from 2.5 to 97.5% face (varied in steps of 5%; Supplementary Fig. 1). After the completion of each block, participants were shown a break screen, and instructed to press a key to continue to the next block. Block order was randomized and counterbalanced across participants. Each block consisted of 80 trials. In each narrow range block, there were 10 morph levels, each presented 8 times. In the wide range, there were 20 morph levels, each repeated 4 times. To adjust for the fact that the overlapping morph range (27.5–72.5% face) that was common to the two blocks included twice as many trials for each morph level in the narrow range block compared to the wide range block, the narrow range was split into a training and test sub-block. The morphs of the training sub-block were shifted up by 0.5%, run first, and not included in the analysis. Accordingly, the test morphs were seen the same number of times in the wide and narrow range blocks, which allowed for a one-to-one comparison for each morph image in the overlapping blocks. Before the start of the first block, participants were given ten practice trials using varying morph percentages from across the wide range, including the common morph range.

#### Experiment 2

Experiment 2 used the same structure, morphing parameters and ranges as Experiment 1. In Experiment 1, participants viewed the overlapping morph levels (presented 240 times in total; 80 times in the two wide range test blocks, 80 times in the two narrow range training blocks, and 80 times in the two narrow range test blocks) three times more than the outer range morphs unique to the wide range (presented 80 times in the wide range test blocks only). To assess whether this difference was driving adaptation effects in Experiment 1, we compensated for it in Experiment 2. Specifically, we tripled the number of outer range morph presentations in Experiment 2, so that every morph level had equal exposure. We also added four trials at the morphing percentage of 50 to determine if there was a preference for faces or houses at the exact middle point in both the narrow and the wide range blocks. This resulted in each wide range block having 124 trials in total relative to the narrow range blocks having 84 trials in total. Experiment 2 was significantly longer than Experiment 1, making it less well suited for fMRI studies, but still feasible for behavioral testing online.

#### Experiment 3

Experiment 3 was conducted to assess whether the results of Experiment 1 replicate in an in-person lab experiment, which would be necessary for a paradigm to be combined with brain imaging (Fig. 2). We used a variant of Experiment 1 with fMRI-compatible timings in the Laboratory for Experimental and Behavioral Economics at the University of Zurich. In this variant, participants were shown the morphed image for 1.3 s. Feedback was given for 1.0 s. Each trial was separated by an average inter-trial interval of 3.5 s, jittered from 1.0 to 9.0 s. Each trial lasted on average 5.8 s; each block lasted 8.3 min.

### Analysis

#### Variables

The main dependent variables were accuracy and response times. Accuracy was quantified as percentage of trials in which participants correctly indicated whether an image was more than 50% face or house as defined by the morph percentage used in creating the image. Response times corresponded to the interval between stimulus presentation and button press. For each participant, we calculated the average accuracy and response time for the full wide range block, the narrow range blocks, as well as for the overlapping morph percentages that were common to the central part of the wide range and the full narrow range blocks. Given that the extreme parts of the wide range are easier to discriminate (for example, 97.5% face is obviously a face, 2.5% face is obviously a house), we expected better overall performance, both in terms of accuracy and response times, in the wide range than in the narrow range.

#### Adaptation score

If participants adapt to the range of most likely morph percentages, they should show improved performance for the same morph percentages (i.e. the overlapping morphs) in the narrow compared to the wide range. To test this prediction, we computed an adaptation score for each participant, which reflected the individual strength of adaptation to the range. For each participant, we determined an accuracy adaptation score:

$${\text{Adaptation score }} = {\text{ mean accuracy }}\left( {\text{overlapping morphs in narrow range condition}} \right) \, - {\text{ mean accuracy }}\left( {\text{overlapping morphs in the wide range condition}} \right).$$

Similarly, we calculated an individual score for response time adaptation by subtracting the mean response times for the overlapping morphs in the narrow range from those in the wide range. Thus, for both the accuracy and response time adaptation score, the higher the score, the stronger the range adaptation.

#### Psychometric functions

We fit a psychometric function to our data using GraphPad Prism, utilizing a sigmoidal fit, separately for the wide and the narrow ranges. Because the sample sizes of individual experiments were limited and the findings converged across experiments, we pooled the data over the three experiments. Data for each individual experiment can be found in the supplemental data file (Supplementary Fig. 2). For each participant we extracted the point of subjective equality (PSE), which corresponds to the estimated morph percentage at which the participant was equally likely to categorize an image as either a face or a house. This allowed us to assess whether participants showed response biases for either face or house and, more importantly, to determine the slope of the psychometric functions. Under adaptation, we expected a steeper slope in the narrow than the wide range, reflecting higher sensitivity to the more limited width of expected inputs.

#### SPQ-BRU

To measure schizotypy in our sample we used the Schizotypal Personality Questionnaire (SPQ)^38^. We utilized this questionnaire to determine the level of schizotypal personality traits in each participant and scaled the responses between 0 and 100% of possible schizotypy score. We added three attention check questions to ensure participants paid attention to the survey. For example, one such item was, “If you are paying attention, choose “1”. Scores are reported as total score and three sub-scores: cognitive perceptual, inter-personal, and disorganized. Our participants’ results fell within the normative range seen in large-scale studies of the non-clinical population using this questionnaire^38–41^.

### Data analysis and Statistics

All demographic, behavioral, and neuropsychological data were analyzed and correlations performed using GraphPad Prism 8, while Bayes factor analysis was conducted using JASP 0.16.3. All analyzed data was tested for normality using the D'Agostino & Pearson test. If there was no significant normality violation, the accuracy data was analyzed with parametric mixed-effects ANOVA or paired *t* test. Conversely, with significant normality violations, the data was analyzed with the appropriate non-parametric test (the uncorrected Dunn's test or the Wilcoxon matched-pairs signed rank test).

## Results

Participants were asked to categorize noisy morphed face-house images as either a face or a house. The images were presented to participants in either a wide range block of morph percentage (2.50–97.5% face; Supplementary Fig. 1), or a narrow range block of morph percentage (27.5–72.5% face), with randomized block order. Each participant completed two wide range blocks and two narrow range blocks.

### Experiment 1

**Figure 3:**
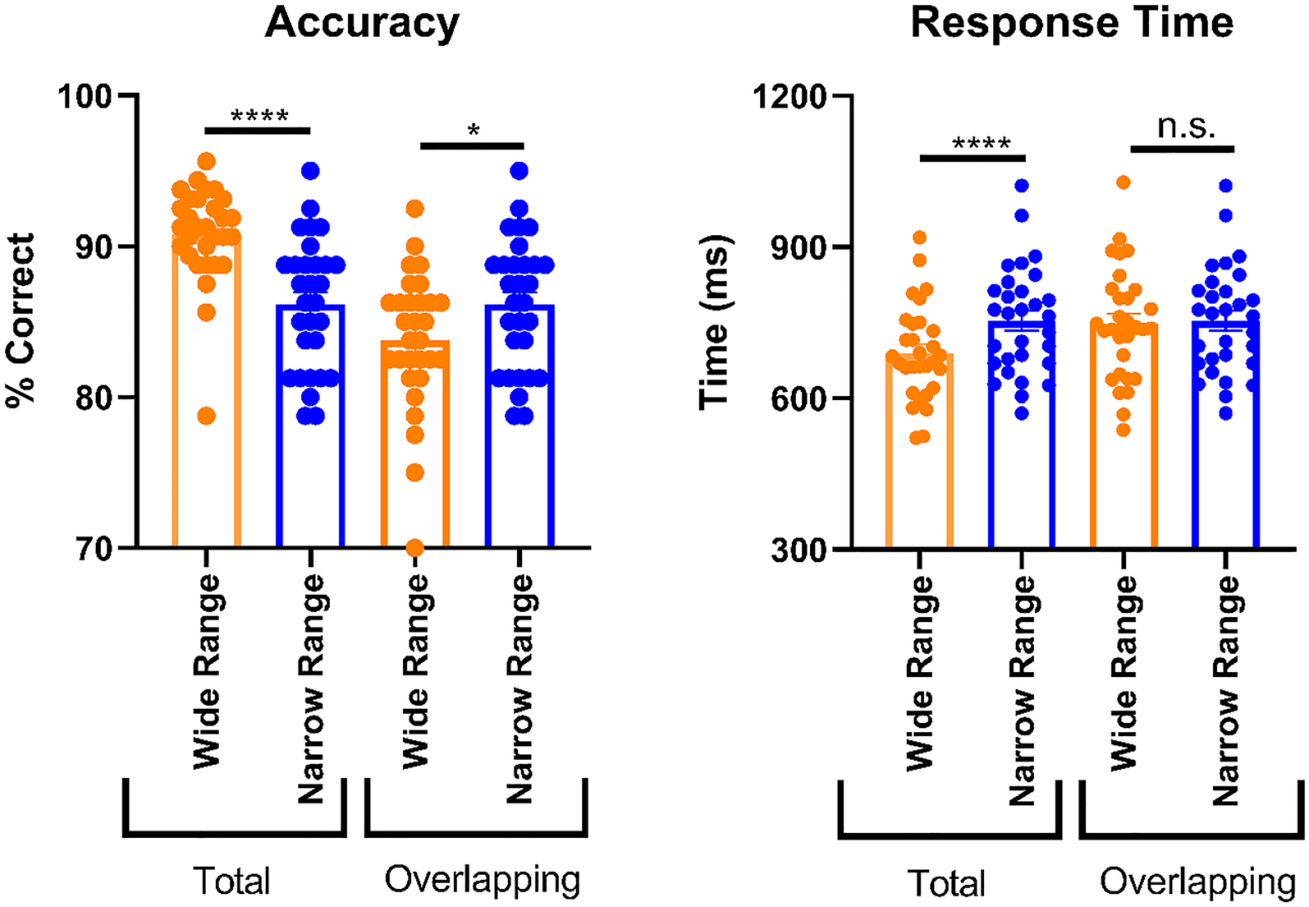
Accuracy and response times from Experiment 1. For accuracy, the percentage (%) correct for the wide and narrow range blocks in the experiment are given. Response time correspond to the interval (ms) from the onset of the stimulus until participants recorded their response. With both measures, the first two bars show the data for the total wide and narrow range blocks, the second two bars for only the overlapping regions of the wide and narrow range blocks. The overlapping regions are the morph percentages that the two range blocks have in common, i.e. 27.5–72.5% face. Please note that the blue data are necessarily identical and that the y-axes do not start at zero. *n.s.* = not significant. * = p < 0.05, **** = p < 0.0001.

To determine the ability of each participant to adapt to the range context, we compared the average accuracies and response times between wide and narrow range blocks. Crucially, we also compared accuracies and response times for the overlapping stimuli (27.5–72.5% face) between the two ranges. In terms of accuracy, we found higher overall performance in the full wide range compared to the full narrow range (higher average accuracy: p < 0.0001, z = 4.35, uncorrected Dunn’s test). We expected this difference because the outer range morphs only present in the wide range condition (i.e. 2.50–22.5% face and 77.5–97.5% face) are particularly easy to categorize as either a face or a house. Importantly, when comparing the average accuracy in the overlapping parts of the two blocks, i.e. the central part of the wide range block with the matched narrow range block, participants were significantly more accurate in the narrow range block (higher average accuracy: p = 0.011, z = 2.55, uncorrected Dunn’s test; Fig. 3). Thus, humans can adapt to range information not only in the reward domain^25,26^ but also in the domain of high-level perception.

In terms of response times, participants were quicker in the full wide range than in the full narrow range (faster average response times: p < 0.0001, t(29) = 5.56, mean difference (MD) 64.53, standard error of the mean (SEM) 11.60, paired *t* test). Again, this effect is expected because the full wide range includes relatively easier trials than the full narrow range. In contrast to accuracy, response times showed little evidence for range adaptation for the morph percentages that were common to the two blocks (faster average response times: p = 0.63, t(29) = 0.48, MD 6.201, SEM 12.69, paired *t* test; Fig. 3). Together, in our object discrimination task, primarily accuracy appears to be sensitive to range information.

### Experiment 2

Experiment 1 showed behavioral range adaptation with unequal exposure to the common morph percentages. Specifically, because Experiment 1 kept the trial number constant for the narrow and wide range blocks, participants viewed the overlapping morph percentages (27.5–72.5% face) more frequently than the outer range morph percentages (2.50–22.5% face and 77.5–97.5% face). To test whether this design feature drove adaptation, we modified the wide range block in Experiment 2 so that each morph percentage was presented the same number of times across the whole experiment, resulting in 124 trials in the wide range block relative to 84 trials in the narrow range block.

**Figure 4:**
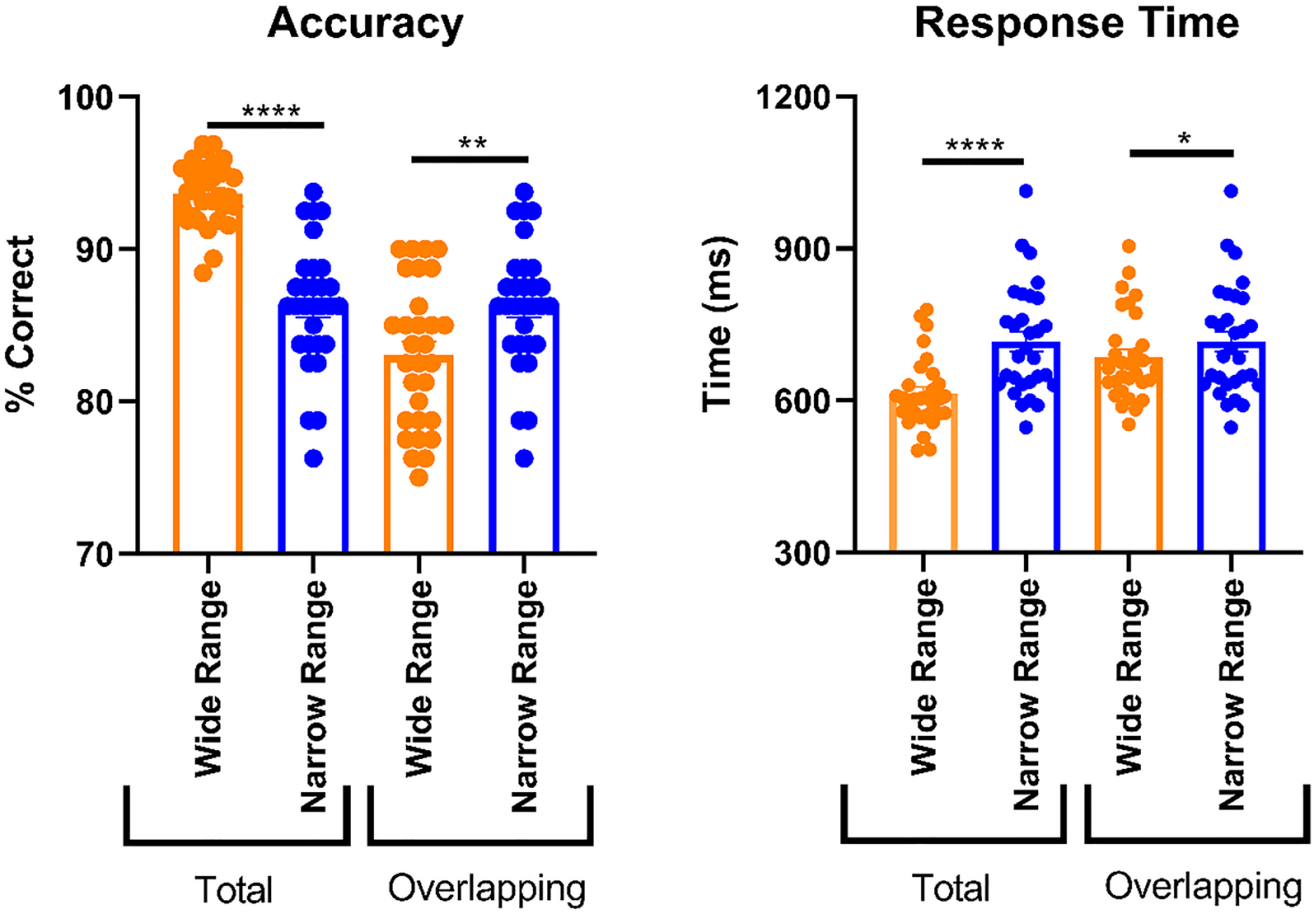
Accuracy and response times from Experiment 2. Conventions as for Experiment 1 (see Fig. 3). * = p < 0.05, ** = p < 0.01, **** = p < 0.0001.

Overall performance in the full wide range block was again significantly higher than in the full narrow range block (higher average accuracy: p < 0.0001, t(29) = 10.28, MD 7.366, SEM 0.7169, paired *t* test; faster average response times: p < 0.001, t(29) = 7.07, MD 102.7, SEM 14.53, paired *t* test). Responses were faster in the wide range block even when restricting analysis to the morphs that overlapped with the narrow range (p = 0.030, t(29) = 2.272, MD 31.60, SEM 13.91, paired *t* test). More importantly, average accuracy was again significantly increased in the narrow range relative to the overlapping wide range (p = 0.007, t(29) = 2.884, MD 3.250, SEM 1.127, paired *t *test; Fig. 4). Thus, Experiment 2 shows range adaptation in object categorization also when the common stimuli are presented as often as the unique stimuli of the wide range, suggesting that the effect observed in Experiment 1 cannot be simply explained by differences in presentation frequency.

Next, we asked whether adaptation strength differed between Experiments 1 and 2. We conducted a mixed effects ANOVA on the accuracy for the overlapping narrow and wide ranges, with Experiment as the between subject factor. We found neither a significant effect of Experiment (F(1,58) = 0.11, p = 0.74, mixed effects ANOVA) nor a significant interaction of Experiment with Range (F(1,58) = 0.43, p = 0.52, mixed effects ANOVA). These findings provide little evidence for differences in adaptation strength between Experiments 1 and 2.

### Experiment 3

Experiment 3 tested whether the findings of online Experiment 1 replicated in-person. We chose to replicate Experiment 1 rather than Experiment 2 because compared to Experiment 1, Experiment 2 did not show stronger adaptation effects but lasted longer. Moreover, we adapted the timings of Experiment 3 for future fMRI experiments.

**Figure 5:**
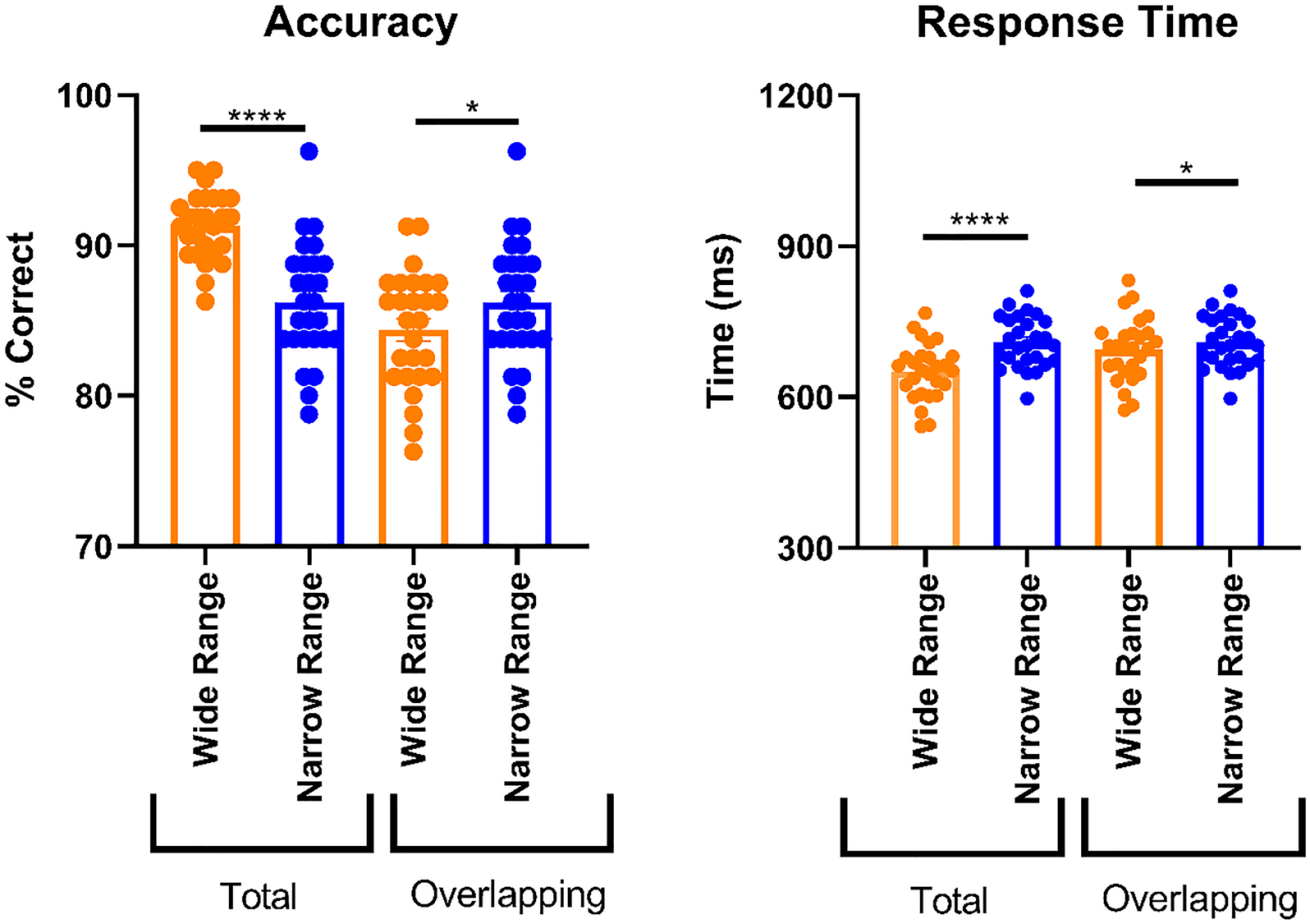
Accuracy and response times from Experiment 3. Conventions as for Experiment 1 (see Fig. 3). * = p < 0.05, **** = p < 0.0001.

Similar to Experiments 1 and 2, overall performance was significantly better in the full wide range than in the full narrow range (higher average accuracy: p < 0.0001, t(26) = 7.250, MD 5.096, SEM 0.703, paired *t* test; faster response times: p < 0.0001, t(26) = 10.43, MD 58.66, SEM 5.624, paired *t* test). As in Experiment 2, responding was faster in the overlapping wide range than in the narrow range (p = 0.027, t(26) = 2.347, MD 14.61, SEM 6.223, paired *t* test). Importantly, we replicate the finding of increased average accuracy in the narrow range compared to the overlapping wide range (p = 0.042, t(26) = 2.14, MD 1.806, SEM 0.842, paired *t* test; Fig. 5). Thus, range adaptation in object categorization occurred also in the lab.

### Psychometric functions

To determine whether participants showed a bias towards face or house perception^21^, we determined the points of subjective equality (PSE) in the psychometric functions in the overlapping morphs across all participants and experiments, illustrated in an averaged psychometric function curve Across all participants, in the narrow range block we found no evidence for bias in favor of face or house By contrast, in the wide range block, participants on average were slightly more likely to categorize ambiguous stimuli as a house than a face When comparing the PSE in the narrow range against the PSE in the wide range for each participant, we found a significant difference (p = 0.035, Sum of signed ranks (W) (87) = − 995.0, Median − 0.010, wide range interquartile range (IQR) = 0.0399, narrow range IQR = 0.0331, Wilcoxon matched-pairs signed rank test). These results indicate that participants were more accurate in their object determinations in the narrow range, indicating a context-specific shift in accuracy.

**Figure 6:**
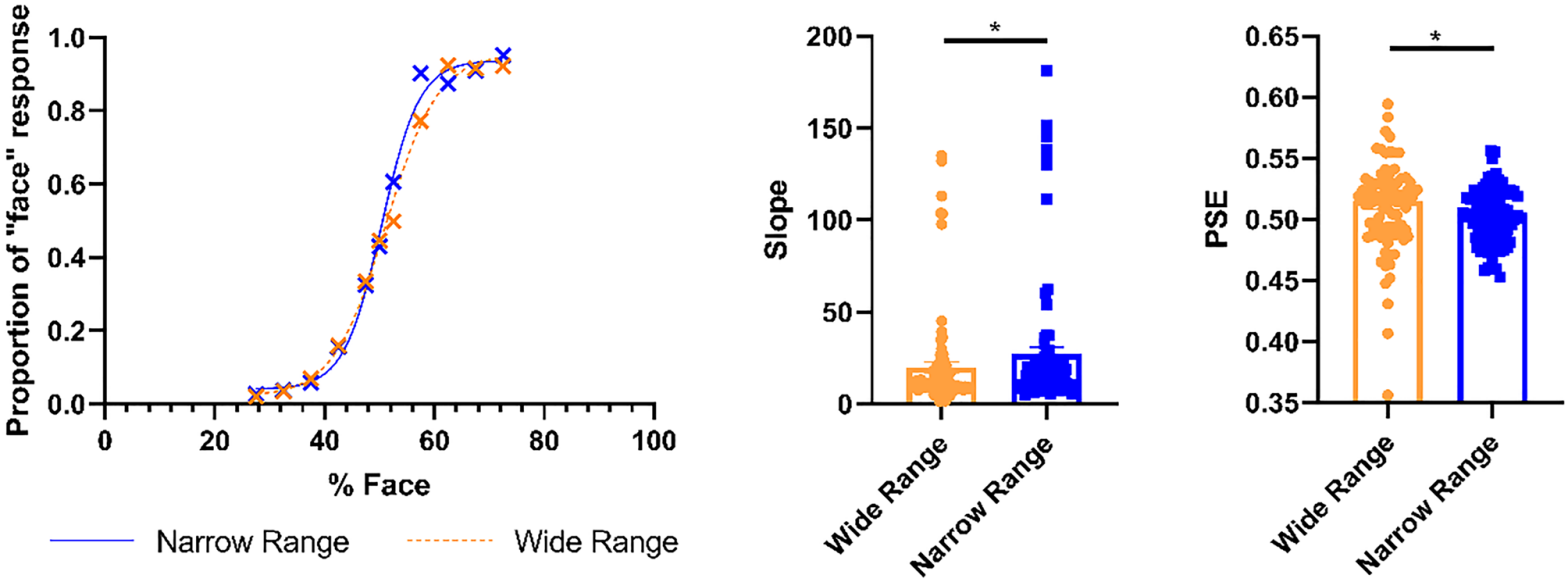
Psychophysical data for the three experiments pooled. Average psychometric functions and individual slopes and points of subjective equality (PSE) are shown separately for the wide and narrow range blocks. We plot the proportion of “face” responses for each face level in the overlapping region (27.5–72.5% face) for narrow and wide range blocks. Crosses show the choice probabilities and lines show the psychometric function fit to the data. * = p < 0.05.

As hypothesized, the slope of the psychometric function for the narrow range blocks was steeper than the slope of the psychometric function for the wide range blocks when comparing the overlapping range. When comparing the slope of the psychometric function for each participant in the narrow range to the wide range, and we found a significant difference (p = 0.0277, W(87) = 1038, Median 2.353, wide range IQR = 8.842, narrow range IQR = 12.10, Wilcoxon matched-pairs signed rank test, Fig. 6). Thus, participants were more sensitive to face and house information in the narrow compared to the wide range block, suggesting that they adapted to the different ranges.

### Relation to schizotypy

To investigate the relation between schizotypy and range adaptation in object categorization, we had participants complete the SPQ-BRU questionnaire after completing the tasks. Previous research has demonstrated a correlation between the level of schizotypal personality traits in the healthy controls and adaptive coding in a reward task^26^. However, this relation remained unexplored in other domains.

**Figure 7:**
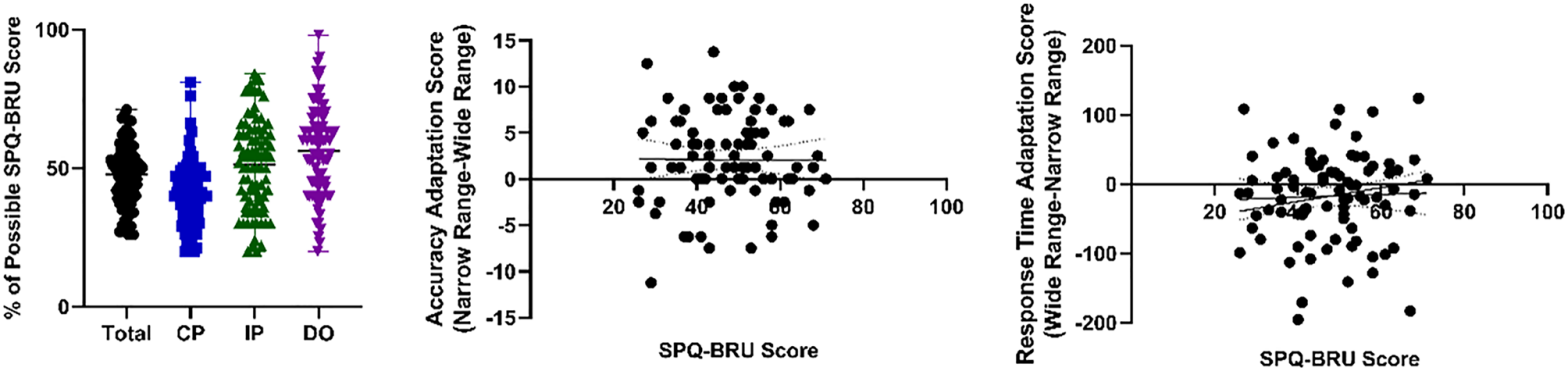
SPQ-BRU scores and correlation with accuracy and response time adaptation scores. We pooled the SPQ-BRU (schizotypal personality questionnaire—brief revised updated) scores of the participants in all three experiments. We display both total scores as well as the sub-scale scores (*CP* cognitive perceptual, *IP* interpersonal, *DO* disorganized). The scatter plots illustrate the relation of total SPQ-BRU score with the accuracy and response time adaptation scores (higher levels in both adaptation scores reflect stronger adaptation to the narrow compared to the wide range).

We found a wide range of SPQ-BRU scores in our sample of participants, with total SPQ-BRU scores of 26–71%: cognitive perceptual subscale scores of 20–81%, inter-personal subscale scores of 20–84%, and disorganized subscale scores of 20–98%. However, we found little evidence for an association between schizotypy and the accuracy (r^2^ = 0.001, slope = − 0.015, p = 0.74, simple linear regression) or response time (r^2^ = 0.001, slope = 0.21, p = 0.74, simple linear regression; Fig. 7) adaptation score within our sample. We also found no associations between the schizotypy subscale scores and accuracy or response time adaptation (Supplementary Fig. 3). To interrogate the relation between composite schizotypy and adaptation in more detail, we turned to Bayes factor analysis^42^. The null hypothesis would postulate that there is no correlation between SPQ-BRU scores and accuracy or response time adaptation scores, while our alternative hypothesis expects a negative correlation between schizotypy and both accuracy and response time adaptation. For both accuracy (BF_10_ = 0.177) and response time (BF_10_ = 0.105) adaptation there was moderate evidence in favor of the null hypothesis. Therefore, our findings provide little evidence for a relation between the ability to range adapt in object categorization and schizotypal personality traits. However, it is also possible that our tasks were not sensitive enough to detect such a relation, or that the relation would only become pronounced at the highest levels of schizotypy or at the neural level.

## Discussion

Our experiments assessed range adaptation for object categorization with a novel variant of a classic task. Across different designs, we find that participants indeed adapt to the range of face/house morphing in which they view the images, as evidenced by improved accuracy in the narrow range condition for the stimuli that overlapped between the two ranges. We found little evidence for a relation between range adaptation and schizotypy in our experiments and sample of participants.

Our findings converge with previous research showing range adaptation in healthy participants in the reward domain and extend it into the domain of high-level perception. For example, the neural activity of reward regions such as the caudate ramped up less steeply in a wide range of reward magnitudes and more steeply in a narrow range^23,25,26^. At the behavioral level, similar conclusions hold for the slopes of reward value judgments^27^ and for error rates in a reward learning paradigm^29^. Our findings indicate that higher forms of perception (and by implication different brain areas) also rely on range adaptation.

Adaptation occurred within relatively short blocks (i.e. 80 trials), which were counterbalanced. Thus, adaptation was present even though a wide block could precede and follow a narrow block. Accordingly, adaptation to face/house ranges appears to be a relatively fast process requiring little exposure to the range, at least with the type of stimuli we used. This finding is in line with previous studies which showed adaptation within blocks of as little as 48^23^ or 62^29^ trials. The relatively quick appearance of adaptation may also explain why Experiment 2 (increased exposure to the extreme parts of the range in the wide range condition) found similar levels of adaptation as Experiment 1. In any case, the adaptation effects observed in Experiments 1 and 3 are unlikely due to increased exposure frequency to the overlapping range.

We observed an improvement in accuracy for the overlapping narrow range stimuli, but no improvement in response times. Indeed, contrary to the hypothesis of adaptation, response times were even slower in the narrow range than in the overlapping wide range in two of the three experiments. These findings are compatible with a speed-accuracy trade-off, but this possibility would need further investigation. We note though that response times might not necessarily be a reliable marker of range adaptation. For example, Haarsma and colleagues found no difference in response times between the range conditions, while observing improved accuracy in the narrow range^29^. It is also possible that adaptation in response times takes longer than adaptation in accuracy–a hypothesis that future studies may want to test.

Our participants showed a mild bias for house responses in the wide range blocks, similar to the trend seen in a previous study using the same stimuli^21^. It is tempting to speculate that the lack of a house bias in the narrow range blocks could reflect range adaptation, such that participants are able to match their subjective perception more accurately to the actual inputs in this context. In any case, the steeper slope of the psychometric functions in the narrow range relative to the wide range indicated a range-adaptive effect. Thus, our three experiments converge on the conclusion that range adaptation can occur for object categorization and is open to study in the lab.

We explored whether participants scoring high on schizotypy would show reduced range adaptation. This aspect of our study was inspired by previous work reporting reduced adaptation to reward range in both healthy participants with high schizotypy and patients with schizophrenia^26^. In disagreement with the notion of domain generality, we find little evidence for relations between schizotypy scores and range adaptation in our participants. However, some caution is required regarding this comparison, because dysfunctional adaptive coding of reward was observed mainly on the neural level and in a sample selected for very high schizotypy. In this study, we defined schizotypy by the SPQ-BRU score which may not capture the entire picture of the schizotypy spectrum in the non-clinical population. It is possible that the use of other schizotypy scales could indeed find a correlation between measured schizotypy and range adaptation. Other scales to consider for future studies would be the Comprehensive Assessment of At-Risk Mental States^43^ or the Structured Interview for Prodromal Syndromes^44^.

Interestingly, recent clinical work also reported mixed findings concerning domain generality. For example, Wang and colleagues found over-adaptation to the range in a group of chronic patients with schizophrenia, but reduced adaptation in a group of first episode psychosis patients^27^. Moreover, Haarsma and colleagues found intact range adaptation in participants with schizotypy but impaired adaptation in patients with first-episode psychosis^29^. The high schizotypy group in that study included help-seeking individuals who potentially were more affected than our participants were. Thus, even participants on the extreme end of the healthy psychosis spectrum show intact range adaptation for some functions. While there is currently little evidence for domain generality on the behavioral level, this may not necessarily apply to the level of neural adaptation and further functional imaging studies are needed to address this point.

## Limitations

Several limitations of our study should be noted. First, the sample sizes for our experiments are relatively small, although our study was appropriately powered. Future studies should replicate the results with a larger sample. Second, due to the fact that we excluded participants with DSM-V Axis I diagnoses, our results are limited to the healthy sub-group of the sample. Future work could use less stringent exclusion criteria to investigate the effect of such diagnoses on adaptive coding. Additionally, for this study we did not recruit by SPQ-BRU scores, therefore we had limited numbers of participants on the very high and very low end of the schizotypy range. Finally, the SPQ-BRU is a useful tool for research purposes, but cannot fully replace evaluation by an experienced clinician for in-person assessment of schizotypy.

## Conclusions

The present work shows range adaptation in the domain of object categorization in the healthy population, both online and in the lab. We find that in this task, range adaptation is not reduced in healthy participants with higher levels of schizotypy.

## Supplementary Information

Supplementary Figures.

## Data Availability

The authors confirm that the data supporting the findings of this study are available within the article and its supplementary material. Additional data queries may be directed to the corresponding author.

## References

[1] Wen B (2012). Time course of dynamic range adaptation in the auditory nerve. J. Neurophysiol..

[2] Kohn A (2007). Visual adaptation: Physiology, mechanisms and functional benefits. J. Neurophysiol..

[3] Krekelberg B, Boynton GM, van Wezel RJ (2006). Adaptation: From single cells to BOLD signals. Trends Neurosci..

[4] Wark B, Lundstrom BN, Fairhall A (2007). Sensory adaptation. Curr. Opin. Neurobiol..

[5] Dai J, Wang Y (2018). Contrast coding in the primary visual cortex depends on temporal contexts. Eur. J. Neurosci..

[6] Jin DZ (2005). Tilt aftereffect and adaptation-induced changes in orientation tuning in visual cortex. J. Neurophysiol..

[7] Seymour KJ (2018). Cortical suppression in human primary visual cortex predicts individual differences in illusory tilt perception. J. Vis..

[8] Seymour K (2013). Altered contextual modulation of primary visual cortex responses in schizophrenia. Neuropsychopharmacology.

[9] Koshiyama D (2020). Reduced auditory mismatch negativity reflects impaired deviance detection in schizophrenia. Schizophr. Bull..

[10] Kirschner M (2016). Deficits in context-dependent adaptive coding of reward in schizophrenia. NPJ Schizophr..

[11] Tobler PN, Fiorillo CD, Schultz W (2005). Adaptive coding of reward value by dopamine neurons. Science.

[12] Burke CJ (2016). Partial adaptation of obtained and observed value signals preserves information about gains and losses. J. Neurosci..

[13] Diederen KM (2017). Dopamine modulates adaptive prediction error coding in the human midbrain and striatum. J. Neurosci..

[14] Freedman DJ (2001). Categorical representation of visual stimuli in the primate prefrontal cortex. Science.

[15] Levine SM, Schwarzbach JV (2018). Cross-decoding supramodal information in the human brain. Brain Struct. Funct..

[16] Woolgar A (2011). Adaptive coding of task-relevant information in human frontoparietal cortex. J. Neurosci..

[17] Loose LS (2017). Switch-independent task representations in frontal and parietal cortex. J. Neurosci..

[18] Furl N (2016). Facial-attractiveness choices are predicted by divisive normalization. Psychol. Sci..

[19] McHugh C (2022). Moral Judgment as Categorization (MJAC). Perspect. Psychol. Sci..

[20] Gutnisky DA, Dragoi V (2008). Adaptive coding of visual information in neural populations. Nature.

[21] Fleming SM (2010). Effects of category-specific costs on neural systems for perceptual decision-making. J. Neurophysiol..

[22] Garcia-Lazaro JA (2007). Shifting and scaling adaptation to dynamic stimuli in somatosensory cortex. Eur. J. Neurosci..

[23] Cox KM, Kable JW (2014). BOLD subjective value signals exhibit robust range adaptation. J. Neurosci..

[24] Louie K, Glimcher PW (2012). Efficient coding and the neural representation of value. Ann. N. Y. Acad. Sci..

[25] Kirschner M (2016). Deficits in context-dependent adaptive coding of reward in schizophrenia. NPJ Schizophr..

[26] Kirschner M (2018). Deficits in context-dependent adaptive coding in early psychosis and healthy individuals with schizotypal personality traits. Brain.

[27] Wang LL (2021). Range-adaptive value representation in different stages of schizophrenia: A proof of concept study. Schizophr. Bull..

[28] Northoff G, Mushiake H (2020). Why context matters? Divisive normalization and canonical microcircuits in psychiatric disorders. Neurosci. Res..

[29] Haarsma J (2021). Precision weighting of cortical unsigned prediction error signals benefits learning, is mediated by dopamine and is impaired in psychosis. Mol. Psychiatry.

[30] Heekeren HR (2004). A general mechanism for perceptual decision-making in the human brain. Nature.

[31] Kanwisher N, McDermott J, Chun MM (1997). The fusiform face area: A module in human extrastriate cortex specialized for face perception. J. Neurosci..

[32] McCarthy G (1997). Face-specific processing in the human fusiform gyrus. J. Cogn. Neurosci..

[33] Wang L (2019). Individual face- and house-related eye movement patterns distinctively activate FFA and PPA. Nat. Commun..

[34] Guo B, Meng M (2015). The encoding of category-specific versus nonspecific information in human inferior temporal cortex. Neuroimage.

[35] Fleming SM (2010). Effects of category-specific costs on neural systems for perceptual decision-making. J. Neurophysiol..

[36] Fleming SM, Huijgen J, Dolan RJ (2012). Prefrontal contributions to metacognition in perceptual decision making. J. Neurosci..

[37] Calvo MG, Lundqvist D (2008). Facial expressions of emotion (KDEF): Identification under different display-duration conditions. Behav. Res. Methods.

[38] Davidson CA, Hoffman L, Spaulding WD (2016). Schizotypal personality questionnaire–brief revised (updated): An update of norms, factor structure and item content in a large non-clinical young adult sample. Psychiatry Res..

[39] Callaway DA (2014). Schizotypal Personality Questionnaire-Brief Revised: Psychometric replication and extension. Personal. Disord..

[40] Kállai J (2018). Schizotypy Personality Questionnaire Brief Revisited (SPQ-BR) Hungarian adaptation and interpretation of factors. Psychiatr. Hung.

[41] Cohen AS (2010). Toward a more psychometrically sound brief measure of schizotypal traits: Introducing the SPQ-brief revised. J. Personal. Disord..

[42] van Doorn J (2021). The JASP guidelines for conducting and reporting a Bayesian analysis. Psychon. Bull. Rev..

[43] Yung AR (2005). Mapping the onset of psychosis: The comprehensive assessment of at-risk mental states. Aust. N. Z. J. Psychiatry.

[44] Miller TJ (2003). Prodromal assessment with the structured interview for prodromal syndromes and the scale of prodromal symptoms: predictive validity, interrater reliability and training to reliability. Schizophr. Bull..

