# Supplementary material for: Adaptive coding occurs in object categorization and may not be associated with schizotypal personality traits
Source: Sci Rep. 2022 Nov 12;12:19385. doi: 10.1038/s41598-022-24127-3 (PMC9653375; doi:10.1038/s41598-022-24127-3)
Supplement: Supplementary file 1 — Supplementary Figures. [file 41598_2022_24127_MOESM1_ESM.docx]

***Adaptive Coding occurs in Object Categorization and is not associated with Schizotypal Personality Traits - Supplementary Information***


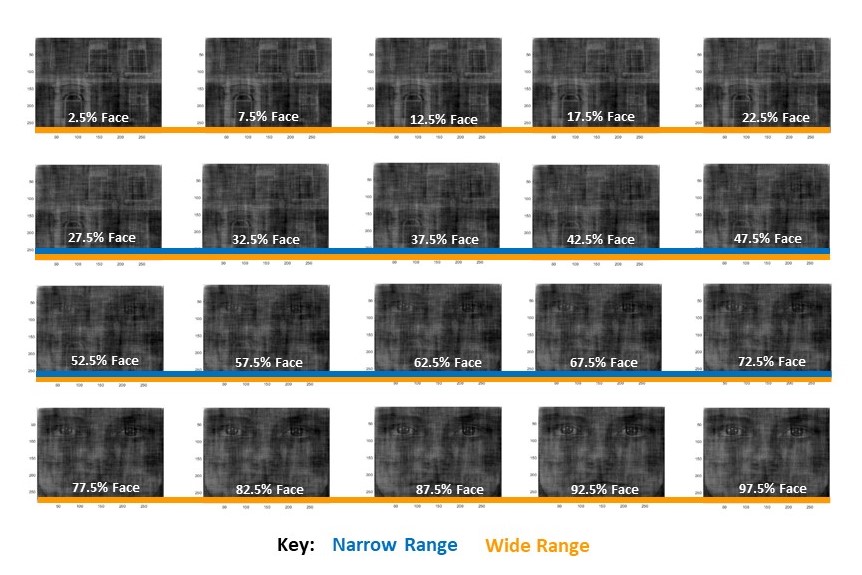


**Supplemental Figure 1. Example face-house stimuli along the morphing continuum.** The wide range is labelled orange, the narrow range blue. The overlapping regions are in the center of the figure, highlighted by both blue and orange.


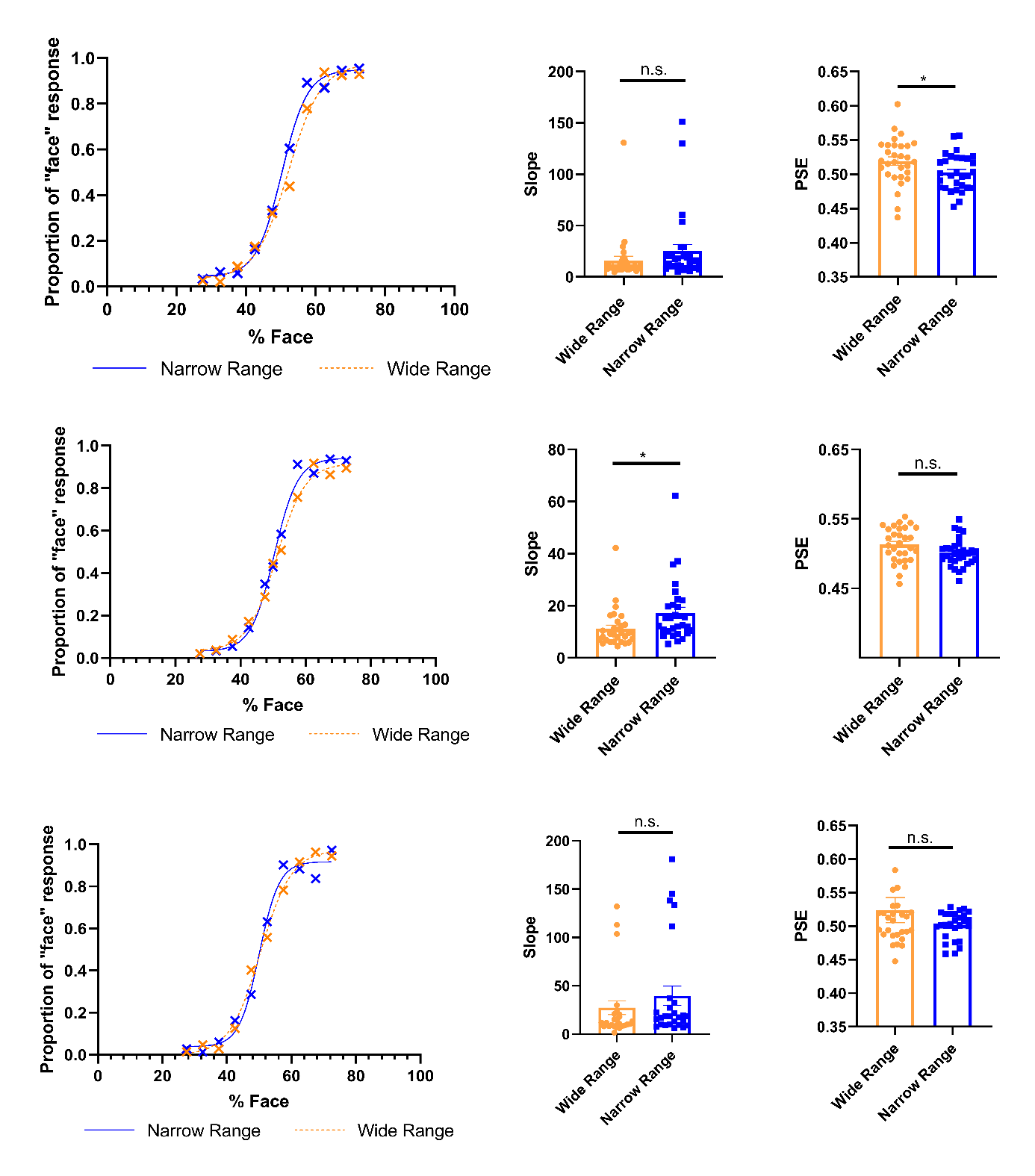


**Supplemental Figure 2.** **Psychophysical data for the three experiments separately.** Average psychometric functions and individual slopes and points of subjective equality (PSE) are shown separately for the wide and narrow range blocks. We plot the proportion of “face” response for each face level in the overlapping region (27.5-72.5 % face) for narrow and wide range blocks. Crosses show the choice probabilities and lines show the psychometric function fit to the data. n.s. = not significant, *= p<0.05


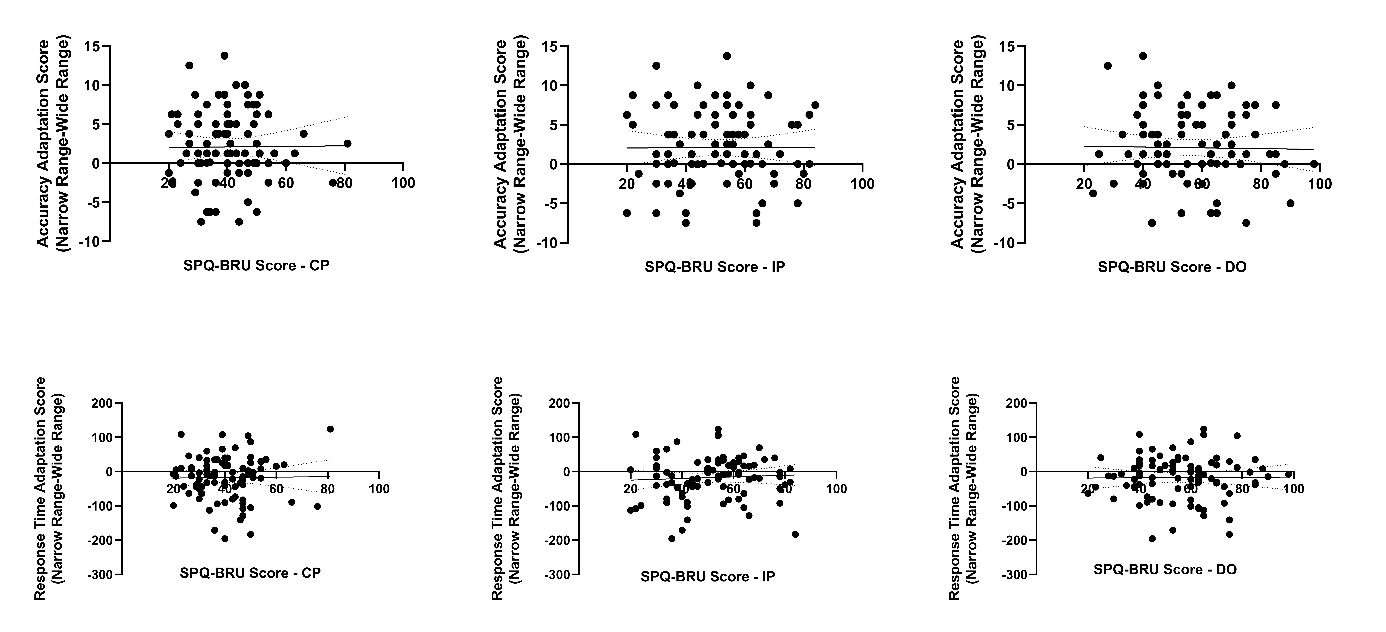


**Supplemental Figure 3.** **SPQ-BRU subscale scores and correlation with accuracy and response time adaptation scores**. We pooled the SPQ-BRU (schizotypal personality questionnaire – brief revised updated) scores of the participants in all three experiments. The sub-scale scores of the SPQ-BRU are the Cognitive Perceptual(CP), the Interpersonal (IP), and the disorganized(DO). These scatter plots illustrate the relation between the SPQ-BRU subscale scores with the accuracy and response time adaptation scores (higher levels in both adaptation scores reflect stronger adaptation to the narrow compared to the wide range).
